# Temperature‐dependent lifespan extension is achieved in *miR*‐*80*‐deleted *Caenorhabditis elegans* by NLP‐45 to modulate endoplasmic reticulum unfolded protein responses

**DOI:** 10.1111/acel.14345

**Published:** 2024-09-25

**Authors:** Chunlin Zhao, Jintao Luo, Yuqiang Zhang, Yong Yu

**Affiliations:** ^1^ State Key Laboratory of Cellular Stress Biology, School of Life Sciences, Faculty of Medicine and Life Sciences Xiamen University Xiamen China

**Keywords:** aging, *Caenorhabditis elegans*, endoplasmic reticulum stress, *miR‐80*, NLP‐45

## Abstract

MicroRNA plays a crucial role in post‐transcriptional gene regulation and has recently emerged as a factor linked to aging, but the underlying regulatory mechanisms remain incompletely understood. In this study, we observed lifespan‐extending effects in *miR‐80*‐deficient *Caenorhabditis elegans* at 20°C but not 25°C. At 20°C, *miR‐80* deletion leads to NLP‐45 upregulation, which positively correlates to increased *abu* transcripts and extended lifespan. Supportively, we identified *miR‐80* binding regions in the 5′ and 3’ UTR of *nlp‐45*. As the temperature rises to 25°C, wildtype increases *miR‐80* levels, but removal of *miR‐80* is accompanied by decreased *nlp‐45* expression, suggesting intervention from other temperature‐sensitive mechanisms. These findings support the concept that microRNAs and neuropeptide‐like proteins can form molecular regulatory networks involving downstream molecules to regulate lifespan, and such regulatory effects vary on environmental conditions. This study unveils the role of an axis of *miR‐80*/NLP‐45/UPR^ER^ components in regulating longevity, offering new insights on strategies of aging attenuation and health span prolongation.

## Graphical abstract

At 20°C but not 25°C, *miR‐80* suppresses NLP‐45 by binding to the untranslated regions of *nlp‐45* primary transcripts (left). Removal of *miR‐80* upregulates NLP‐45 and ABU proteins, and activated ABU proteins benefit longevity as noncanonical components to process endoplasmic reticulum stress (right).

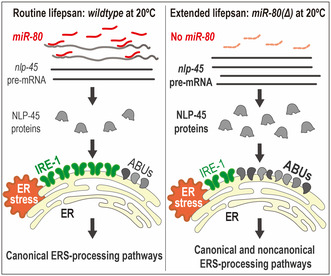

**Abbreviations**
ABU or *abu*
: activated in blocked unfolded protein response
*C. elegans*
: *Caenorhabditis elegans*
ER: endoplasmic reticulum
ERS: endoplasmic reticulum stress
GFP: green fluorescent protein
HSP: heat shock protein
*lf*
: *loss of function*
miRNA(s) or *miR*
: mircoRNA(s)
NLP(s) or *nlp*
: neuropeptide‐like protein
RNAi: ribonucleic acid interference
UPR^ER^
: unfolded protein response, endoplasmic reticulum
UTR: untranslated region

## 1 INTRODUCTION

Aging constitutes a multifaceted phenomenon involving intricate changes within a metabolic network comprising diverse molecules expressed across various tissues in response to variable environmental stimuli (Lopez‐Otin et al., 2023). Pioneering studies in model organisms have demonstrated that aging is influenced by genetic and regulatory mechanisms that respond to nutrient availability and environmental stress (Miller et al., 2020). Activation of CRH‐1/CREB in the AFD thermosensory neurons functions to maintain *C. elegans* lifespan in warm environment (Chen et al., 2016; Lee & Kenyon, 2009), and participations of DAF‐16/FOXO, INS‐7 and several other molecules are known to be important for the regulation of lifespan by temperatures (Lee & Lee, 2022).

Coordination between an animal's external environment and the internal state requires continuous regulation through various regulators including microRNAs (miRNAs) (Ferrante & Conti, 2017). miRNAs are a class of non‐coding single‐stranded RNA molecules encoded by endogenous genes, approximately 19–24 nucleotides in length, which participate in post‐transcriptional gene expression regulation in animals and plants (Zisoulis et al., 2010). Since the discovery of the first microRNA, *lin‐4*, in *C. elegans* (Lee et al., 1993), there has been an increasing number of research findings on the roles of various microRNAs in growth, development, metabolism, stress resistance, and aging. In previous studies, microRNAs have been regarded as effectors that act as molecular sponges, attenuating the impact of stressors such as temperature, pathogens, and ion concentrations on organisms. Many microRNAs can regulate processes including DNA damage response (Wan et al., 2011), mitochondrial metabolism (Yan et al., 2021), and protein homeostasis (Konishi et al., 2021), thereby playing important roles in lifespan regulation.

Neuropeptides are a diverse group of short peptides that primarily bind to similar G protein‐coupled receptors (Hokfelt et al., 2000). Due to this diversity, neuropeptide receptor signaling provides rich multifunctionality in the modulation of neural circuits (Bargmann, 2012; Mirabeau & Joly, 2013). Neuropeptide signaling plays crucial roles in regulating physiological functions such as growth, development, behavior, and reproduction in the nervous system and many other tissues, responding to stimuli from the external environment (Li et al., 2021; Watteyne et al., 2020). Neuropeptide‐like proteins (NLPs) are proteins that share some structural or functional characteristics with neuropeptides, which might be involved in similar biological processes as neuropeptides (Mousley et al., 2010).

Although progress has been made in elucidating the functions of neuropeptides or NLP in lifespan, our understanding of their underlying mechanisms is still limited, and the relationship between miRNA and neuropeptides/NLPs remains unclear. Previous studies have confirmed the important role of miRNA in regulating the lifespan of *C. elegans*, with mechanisms varying depending on the specific miRNA involved (Ambros, 2011; Xu et al., 2019). Therefore, is there a neuropeptide that can serve as a crucial mediator between miRNAs and their downstream targets, enabling organisms to respond to environmental changes that affect lifespan? And would the impact on lifespan vary with changes in the levels of the corresponding neuropeptides?

Here, using *C. elegans* as the model, we found that removal of *miR‐80* extends animals' lifespan at 20°C but shortens it at 25°C, and we identified *miR‐80* binding sites in the 5′ and 3’ UTR of *nlp‐45*, which encodes NLP. Furthermore, we demonstrated that the regulation of lifespan by NLP‐45 or *miR‐80* manifests in the presence of moderate to strong ERS (endoplasmic reticulum stress) via multiple UPR^ER^ components. Our study elucidates the aging‐regulating effect of a temperature‐sensitive axis involving *miR‐80*, NLP‐45, and other ERS processing molecules, and provides a new research interest for investigating the molecular mechanisms of aging.

## 2 RESULTS

### 2.1 Temperature‐dependent effects of *miR* ‐*80(Δ)* on *C. elegans* lifespan

**FIGURE 1:**
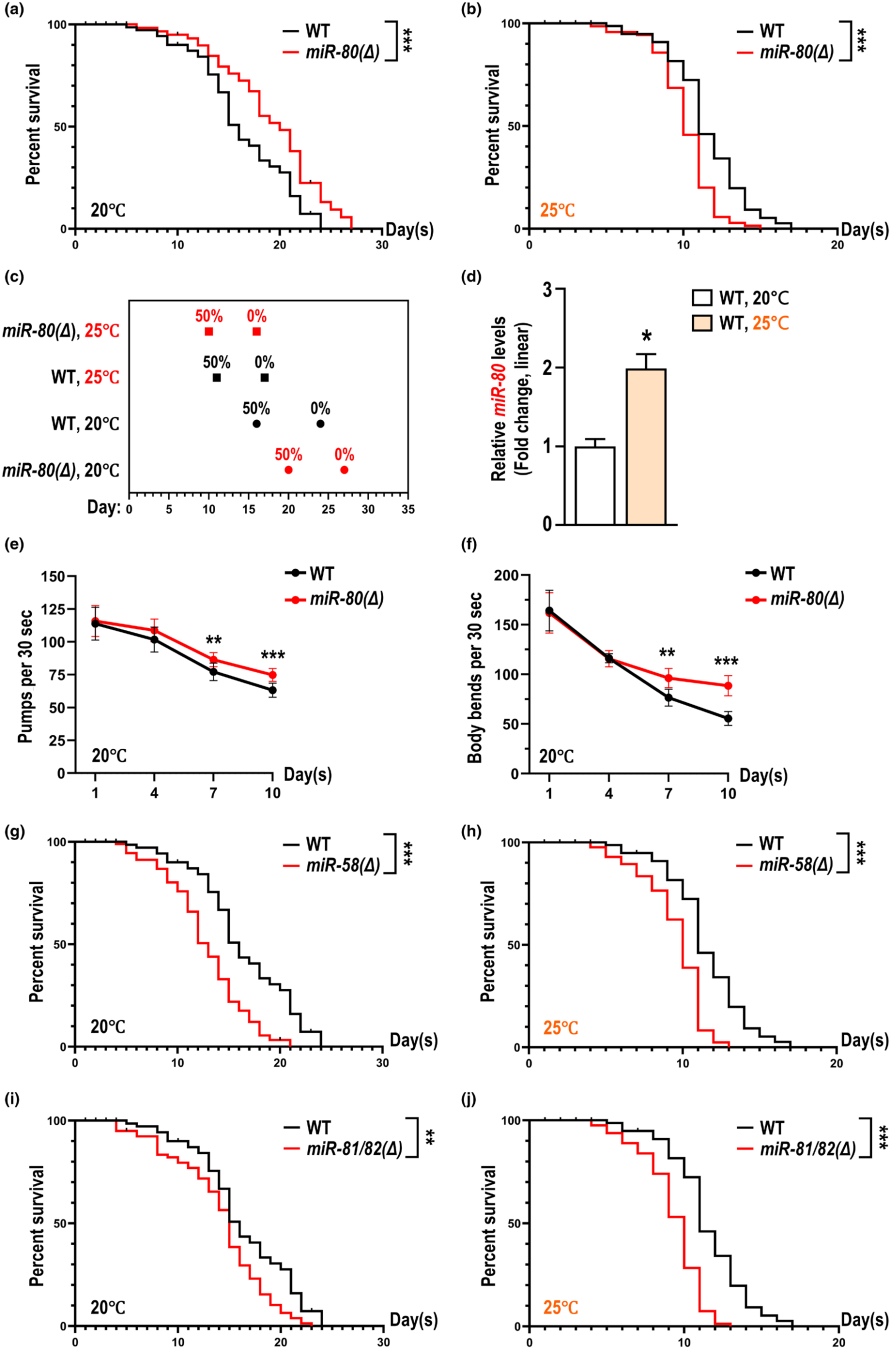
Removal of *miR‐80* extends lifespan and health span in *C. elegans* at 20°C but not 25°C. (a–c) Removal (Δ) of *miR‐80* extends lifespan at 20°C (a), and shortens lifespan at 25°C (b), with exact time points for the survival rate to achieve 50% and 0% being plotted in (c). In all lifespan assays of this article, unless otherwise noted, statistical analyses were processed by log‐rank tests under a null hypothesis assuming the two groups were not different. For all lifespan experiments in this study, even if multiple trials were performed and analyzed for some experiments as in Table S1, for simplicity, only one batch of data was plotted for each experiment. (d) Relative *miR‐80* levels of wildtype cultured at 20°C and 25°C. Linear fold change value was plotted as mean with error bars indicating standard error (s.e.). For all qPCR experiments in this study, Welch's t‐tests were applied under a null hypothesis assuming two groups are not different. (e, f) Counts of an individual animal's pharyngeal pumps (e) and body bends (f) in 30 s at 20°C. Mean and s.e. values were plotted for each time point, and Mann–Whitney tests were applied under a null hypothesis assuming the two groups were not different. (g–j) Lifespan assays with wildtype and mutants lacking coding region of either *miR‐58* (g, h) or *miR‐81* as well as *miR‐82* (i‐j) at 20°C (g, i) or 25°C (h, j). Meanings of statistical symbols of all types of tests in this study share the same rule: One asterisk for *p* < 0.05, two asterisks for *p* < 0.01, three asterisks for *p* < 0.001, and ‘ns’ (not significant) for *p* ≥ 0.05. Animals of the same genotype as in (a) & (b), or (g) & (h), or (i) & (j) were same‐day siblings.

In *C. elegans*, the *miR‐58* family provides the most abundant miRNAs, consisting of members such as *miR‐58*, *miR‐80*, *miR‐81*, and *miR‐82*, with their association primarily based on the similarity of their seed sequences (Zhang et al., 2018). To further understand the differences between members of the *miR‐58* family, we measured the lifespan and health span of mutants lacking miRNA genes of this family. Consistent with Zhang et al (Zhang et al., 2018), at 20°C we confirmed that only the *miR‐80* null mutation, *miR‐80(Δ)*, attenuates aging, while removal of *miR‐58* or *miR‐81/82* accelerates aging (Figure 1a,c,g,i). The health span, indicated by pharyngeal pumping rates and body bends on the 1st, 4th, 7th, and 10th day of adulthood, is also improved in *miR‐80(Δ)* at 20°C (Figure 1e,f), which is consistent with the results of Mehul Vora et al (Vora et al., 2013).

To challenge animals with mild and continuous stress, we assayed lifespan at 25°C, which is permissive for the wildtype *C. elegans* to survive, but with a much shorter lifespan (Figure 1c). *miR‐58* or *miR‐81/82* mutants are short‐lived at both 20°C and 25°C (Figure 1g–j), which is not surprising given their positive regulatory roles in health maintenance (de Lucas et al., 2015; Zhang et al., 2018). For *miR‐80* mutants, intriguingly, lifted temperature abolished the life‐extending effect and completely reversed the viabilities of *miR‐80(Δ)* and control (Figure 1b,c). This phenomenon encouraged us to compare *miR‐80* levels in wildtype cultured at 20°C and 25°C, and we found that *miR‐80* levels are 2‐fold higher at 25°C than at 20°C (Figure 1d), which suggests a possibility for the *miR‐80* molecules to be aging‐accelerating. We excluded a possibility for the levels of *miR‐58/81/82* to be compensatorily upregulated in *miR‐80(Δ)* (Figure S1a,b), so we hypothesize that the aging‐accelerating role of *miR‐80* is unique, distinct from other *miR‐58* family molecules. Specifically, we consider that *miR‐80* could have suppressed the expression of certain life‐extending molecules by pairing with their transcripts for targeted degradation.

*miR‐80(Δ)* fails to extend lifespan at 20°C when *miR‐58* or *miR‐81/82* are simultaneously removed (Figure S1c–e), and no life‐extending effect had been detected at 25°C for *miR‐80(Δ)* on animals lacking *miR‐58/81/82* (Figure S1f–h), so we speculate that the 20°C‐specificity comes from either *miR‐80* per se, or mechanisms downstream of *miR‐80*, or combined. Additionally, we do not exclude the possibility that the lifespan‐extending effect of *miR‐80(Δ)* at 20°C requires functional *miR‐58* and *miR‐81/82*. Together, our findings point to a possibility that one or more targets of *miR‐80* could modulate animals' lifespan and health conditions in a temperature‐dependent manner.

### 2.2 Transcriptome analysis suggesting neuropeptide pathway's role in *miR‐80*‐mediated lifespan regulation

**FIGURE 2:**
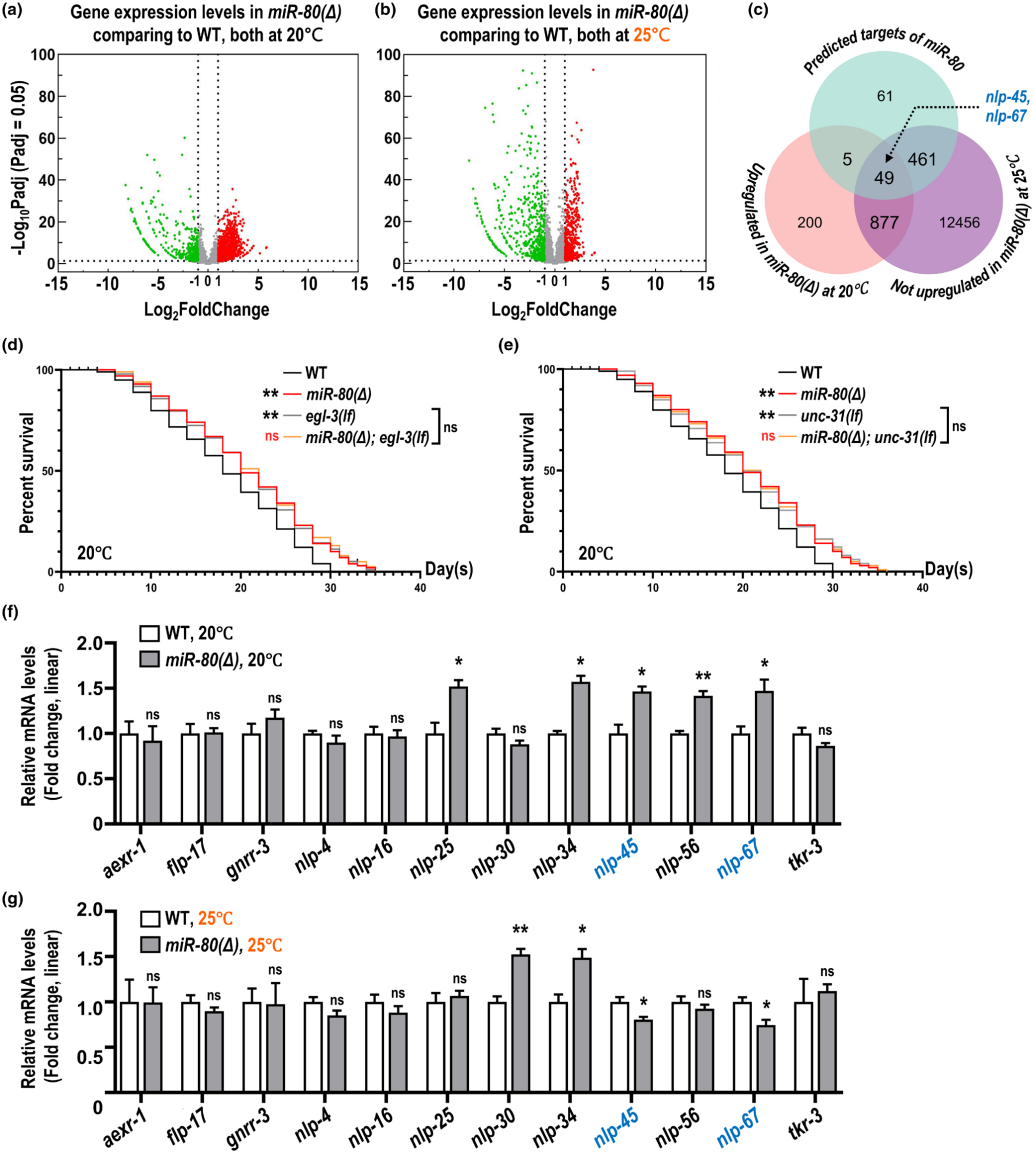
Involvement of neuropeptide pathways in *miR‐80(Δ)‐*mediated lifespan extension. (a, b) Volcano plots illustrate the transcriptomic landscape of *miR‐80(Δ)* compared to wildtype as young adults at 20°C (a) or 25°C (b). Red dots indicate upregulation, and green dots indicate downregulation. (c) Venn diagram plotted by TBtools (Chen et al., 2020) shows specific numbers of genes in each subset, and locations of genes encoding NLP‐45 or NLP‐67 were indicated. (d, e) The lifespan‐extending effect of *miR‐80(Δ)* is suppressed when the neuropeptide pathways are blocked by *egl‐3(lf)* (d) or *unc‐31(lf)* (e). Two black asterisks next to short lines indicate *p* < 0.01 comparing to wildtype by log‐rank test; red ‘ns’ symbols next to short lines indicate *p* ≥ 0.05 comparing to *miR‐80(Δ)* by log‐rank test; black ‘ns’ symbols next to brace symbols indicate *p* ≥ 0.05 in log‐rank test for comparisons between two referred groups represented by their corresponding short lines. (f, g) Linear fold change for the relative transcript levels of genes in neuropeptide pathways by qPCR in *miR‐80(Δ)* and wildtype at 20°C (f) and 25°C (g). Statistics are described in the legends of Figure 1.

To elucidate the temperature‐dependent mechanisms of lifespan regulation by *miR‐80*, we conducted an RNA‐seq analysis to compare transcriptomic profiles of wildtype and *miR‐80(Δ)* animals respectively cultured at 20°C and 25°C. Comparing to wildtype of the same temperature, *miR‐80(Δ)* at 20°C has 482 downregulated genes and 1131 upregulated genes, and *miR‐80(Δ)* at 25°C has 774 downregulated genes and 578 upregulated genes (Figure 2a,b; Table S3). These differences in gene expression under varied temperature conditions prompted us to further explore specific targets of *miR‐80*.

Integrating potential *miR‐80's* target genes identified from the TargetScan database (https://www.targetscan.org/worm_52/) (McGeary et al., 2019) with our RNA‐seq data, we identified neuropeptide‐like protein encoding genes, *nlp‐45* and *nlp‐67*, appear to be not only potential *miR‐80* targets but also with differentially expressed patterns in *miR‐80(Δ)* (Figure 2c). There are other neuropeptide pathway‐related genes, as mentioned in Figure 2f,g, showed expression patterns similar to *nlp‐45*'s in RNA‐seq, although without predicted *miR‐80* binding sites. We performed lifespan assays in *egl‐3(lf)* (Husson et al., 2007) and *unc‐31(lf)* (Charlie et al., 2006), each known to disrupt the maturation or secretion of neuropeptides. At 20°C, deletion of *miR‐80* failed to further extend the lifespan of the already long‐lived *egl‐3(lf)* (Hamilton et al., 2005; Zullo et al., 2019) or *unc‐31(lf)* (Figure 2d,e). Thus, we speculate that certain lifespan‐extending peptides (and/or receptors) were suppressed by *miR‐80* in wildtype, and *miR‐80(Δ)* extends lifespan by the uninhibition of these targets at 20°C.

### 2.3 Regulation of lifespan by *miR‐80* through targeting *nlp‐45*

To pinpoint the specific targets of *miR‐80* implicated in lifespan regulation, we conducted qPCR analysis on the aforementioned neuropeptide pathway genes. Levels of *nlp‐45* and *nlp‐67* indeed increased in *miR‐80(Δ)* at 20°C and decreased in *miR‐80(Δ)* at 25°C (Figure 2f,g). However, removal of *miR‐80* still managed to extend the lifespan in *nlp‐67(lf)*, but not in *nlp‐45(lf)* (Figure S2a,b). Therefore, we shifted our focus to *nlp‐45*.

**FIGURE 3:**
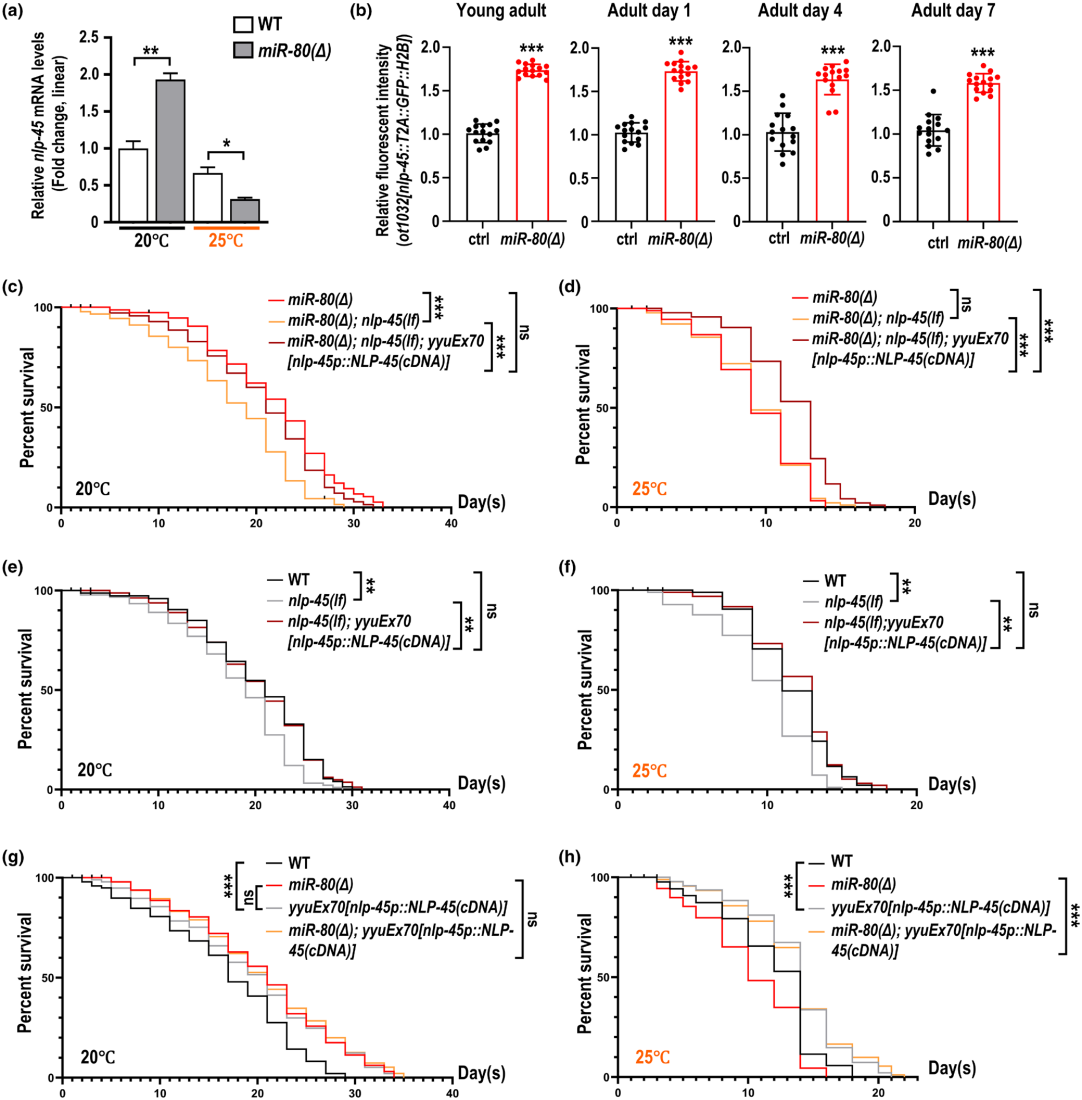
NLP‐45 overexpression extends lifespan at 20°C and 25°C regardless of *miR‐80*'s presence. (a) Transcripts of *nlp‐45* in *miR‐80(Δ)* and wildtype at 20°C or 25°C by qPCR. (b) In the young adult stage and on the 1st, 4th, and 7th day of adulthood, GFP intensities of *ot1032[nlp‐45::T2A::GFP::H2B]* were imaged and compared in between *miR‐80(Δ)* and wildtype at 20°C. Columns indicate the mean values, and error bars indicate s.e. Three asterisks indicate *p* < 0.001 for Mann–Whitney tests under a null hypothesis assuming two groups are not different. Each dot represents an individual animal. (c, e) At 20°C, reintroduction of NLP‐45 via *yyuEx70* restored animals' viability to the controlling strain's level in the background of *miR‐80(Δ); nlp‐45(lf)* (c) or *nlp‐45(lf)* (e). (d, f) At 25°C, *yyuEx70* attenuates aging in age‐prone *miR‐80(Δ); nlp‐45(lf)* (d) or *nlp‐45(lf)* (f). (g, h) Overexpression of NLP‐45 by *yyuEx70* effectively attenuates aging in wildtype or *miR‐80(Δ)* at 20°C (g) and 25°C (h). Statistics are described in the legends of Figure 1. Lifespan assays in (c) & (e), or (d) & (f) were done in a same‐day manner.

In examining the influence of *miR‐80* on NLP‐45, we utilized the GFP knocked‐in NLP‐45 strain OH16380 *nlp‐45(ot1032[nlp‐45::T2A::GFP::H2B])*. In *miR‐80(Δ)* animals, the GFP intensity is generally increased at 20°C across various stages (young adult, the 1st, 4th, and 7th day of adulthood) (Figure 3b), and generally decreased at 25°C (Figure S3), which is consistent to the results of another qPCR on *nlp‐45* with wildtype and *miR‐80(Δ)* templates respectively collected from two temperatures (Figure 3a).

We further analyzed the role of NLP‐45 in regulating lifespan by introducing multi‐copied transgene with the coding region and endogenous promoter of *nlp‐45*, namely *yyuEx70*, into the strains used in Figure S2a. Regardless of *miR‐80*'s presence, at 20°C *yyuEx70* restored the viability of *nlp‐45(lf)* to the control level (Figure 3c,e). In another experiment, overexpressed NLP‐45 extends lifespan as efficiently as *miR‐80(Δ)* does, while additive lifespan‐extension effect cannot be detected for *yyuEx70* and *miR‐80(Δ)* (Figure 3g). At 25°C, a condition with primitively fewer *nlp‐45* transcripts (Figure 3a), we found that introduction of *yyuEx70* benefits longevity in *nlp‐45(lf)* (Figure 3f), *miR‐80(Δ)* (Figure 3h) and *miR‐80(Δ); nlp‐45(lf)* (Figure 3d), suggesting a likelihood of *nlp‐45* functioning independent or downstream of *miR‐80* to improve survival. As we confirmed that the *nlp‐45* levels are indeed lowered in *miR‐80(Δ)* at 25°C (Figures 2g and 3a; Figure S3), we speculate that a mysterious factor, X, may downregulate *nlp‐45* and outweigh *miR‐80*'s impact on *nlp‐45* at this temperature (Figure 6i and the Discussion).

### 2.4 Direct regulatory control of *miR‐80* on *nlp‐45* expression

**FIGURE 4:**
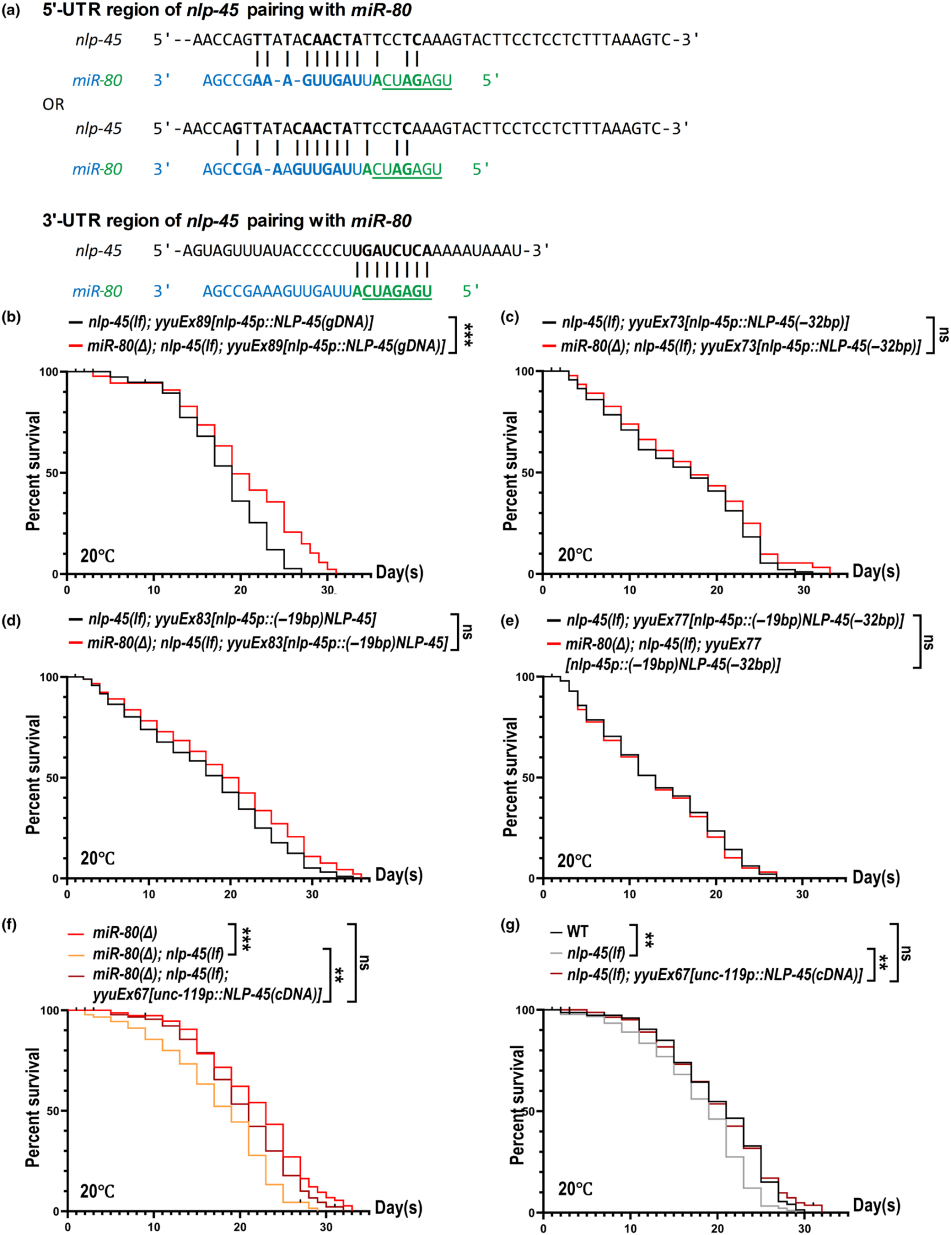
*miR‐80* directly regulates *nlp‐45* at its UTR sites to regulate aging. (a) Predicted binding sites of *miR‐80* on the 5’‐UTR and 3’‐UTR of *nlp‐45*. *miR‐58* family's seed region has been underlined. (b) *miR‐80(Δ)* extends lifespan of *nlp‐45(lf)* when NLP‐45 is reintroduced with proper UTR sites. (c–e) *miR‐80(Δ)* fails to extend the lifespan of *nlp‐45(lf)* when NLP‐45 is reintroduced without proper UTR sites, either lacking the 3’‐UTR namely (−32 bp) (c), or 5’‐UTR namely (−19 bp) (d), or both (e). (f, g) Pan‐neural overexpression of NLP‐45 restored the lifespan of *nlp‐45(lf)* to the controlling strain's level, with or without functional *miR‐80*. Statistics are described in the legends of Figure 1.

miRNAs usually regulate gene expression by binding to the target gene's primary transcripts at 3′‐ or 5’‐UTR sites (O'Brien et al., 2018; Wongfieng et al., 2017). To determine if there are direct interactions between *miR‐80* and NLP‐45, we analyzed the complementary properties of *miR‐80* and *nlp‐45* UTR. Indeed, there are multiple sites in the 5′‐ and 3’‐UTR of *nlp‐45* that can directly complementarily pair with *miR‐80* (Figure 4a).

To validate the functionality of these sites, we engineered transgenic strains in *nlp‐45(lf)* background by introducing *nlp‐45* genomic DNA fragments without predicted *miR‐80* binding sites, followed by lifespan analysis. Notably, the reintroduction of *nlp‐45* containing its native 5′‐ and 3’‐UTRs resulted in a significant preservation of the lifespan extension effect for *miR‐80(Δ)* (Figure 4b). In contrast, deletion of *miR‐80* complementary base pairs within the 5′‐ and/or 3’‐UTR of *nlp‐45* led to a notable suppression of this lifespan extension effect (Figure 4c–4e). When the 5’‐UTR binding sites were removed from the multi‐copied *nlp‐45* transgene (*yyuEx83*), animals still survived for a fair amount of time, and *miR‐80(Δ)* may have slightly extended the lifespan of *yyuEx83*, though not statistically significant (Figure 4d). When the 3’‐UTR sites were removed, *miR‐80(Δ)* barely extends lifespan (Figure 4c), and the most pronounced impact on lifespan was observed with alterations to both 5′‐ and 3’‐UTR (Figure 4e). In all these transgenes mentioned above, *nlp‐45* is overexpressed under the condition of *miR‐80(Δ)* (Figure S4a), which is reasonable given that the suppressive effect of *miR‐80* on *nlp‐45* transcripts are eliminated. For worms with functional *miR‐80*, levels of *nlp‐45* transcripts are significantly higher in transgenic animals bearing impaired *miR‐80* binding sites, for example, *yyuEx83*, indicating the incompleteness of *miR‐80*'s inhibitory effects (Figure S4b).

### 2.5 Tissue specificity of NLP‐45

Following the identification of NLP‐45 as a key target of *miR‐80* in lifespan regulation, we sought to pinpoint the specific tissues where NLP‐45 exerts its regulatory effects. Given that NLP‐45 is a neuropeptide‐like protein with neural expression (Liska et al., 2023; Sun & Hobert, 2021), we engineered transgenic strain *yyuEx67[unc‐119p::nlp‐45 cDNA::SL2::GFP]* for pan‐neural overexpression of NLP‐45, aiming to determine if pan‐neural expression of NLP‐45 is sufficient to restore the survival of short‐lived *nlp‐45(lf)*. Indeed, *yyuEx67* restores the lifespan of *miR‐80(Δ); nlp‐45(lf)* to a *miR‐80(Δ)*‐like level, and *yyuEx67* restores the lifespan of *nlp‐45(lf)* to a wildtype‐like level (Figure 4f,g).

Considering that NLP‐45 is expressed earliest in RIA neurons during development (Sun & Hobert, 2021), we wondered if RIA neurons may be primary working sites. Therefore we utilized *otEx7677[mgl‐1p::nlp‐45 cDNA::SL2::TagRFP::p10 3′ UTR + inx‐6(prom18)::TagRFP]*, which specifically reintroduces NLP‐45 in RIA neurons, in lifespan assays. However, the aging‐alleviating effect of RIA‐specific NLP‐45 restoration is partial (Figure S4c,d). This finding indicates that although RIA neurons are the earliest site of NLP‐45 expression, they may not necessarily be the primary site for NLP‐45's aging alleviation. Relatedly, in an experiment described in 2.7, pan‐neural expression of NLP‐45 did not extend lifespan in wildtype treated with tunicamycin (Figure S6e), bringing up complexity for the tissue‐specificity of NLP‐45.

### 2.6 NLP‐45's modulation on *abu* levels

We delved deeper into the downstream effects of NLP‐45 by mining RNA‐seq data as shown in Figure 2a,b. Comparing *miR‐80(Δ)* to wildtype for 20°C‐specifically upregulated genes and 25°C‐specifically downregulated genes, 137 genes overlapped, and 8 of them encode ABU (Activated in Blocked UPR) proteins. ABU proteins are known as noncanonical UPR^ER^ (Unfolded Protein Response, Endoplasmic Reticulum) components, and *abu* transcripts would upregulate upon impaired IRE‐1/XBP‐1 pathway in contexts with strong ERS (Haskins et al., 2008). In *miR‐80(Δ)* cultured at 20°C, upregulated *nlp‐45* and *abu* transcripts, as well as extended lifespan, encouraged us to hypothesize that abundant NLP‐45 may alleviate aging via promoted ABU proteins.

**FIGURE 5:**
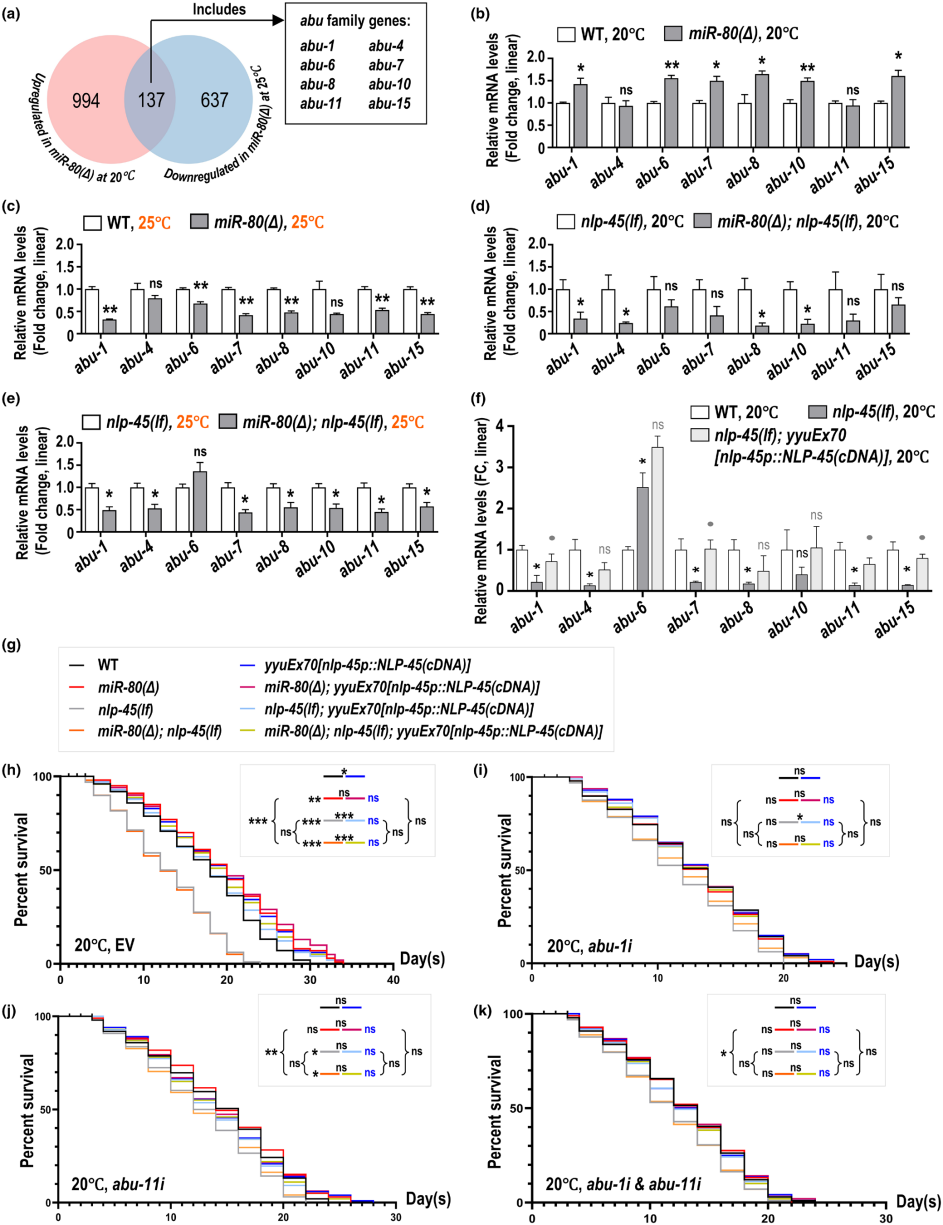
ABU proteins are required for *miR‐80(Δ)* or NLP‐45 overexpression to extend lifespan. (a) Venn diagram plotted with TBtools (Chen et al., 2020) indicating the same expression pattern of 8 *abu* genes as being 20°C‐specifically upregulated and 25°C‐specifically downregulated in *miR‐80(Δ)* controlled by wildtype. (b‐c) Relative transcript levels of qRT‐PCR on *abu* genes in *miR‐80(Δ)* controlled by wildtype at 20°C (b) or 25°C (c). (d, e) Relative transcript levels of qRT‐PCR on *abu* genes in *miR‐80(Δ)*; *nlp‐45(lf)* controlled by *nlp‐45(lf)* at 20°C (d) or 25°C (e). (f) Relative transcript levels of qRT‐PCR on *abu* genes in *nlp‐45(lf)* controlled by wildtype (black asterisks or ‘ns’) and in *nlp‐45(lf)*; *yyuEx70* controlled by *nlp‐45(lf)* (grey dots or ‘ns’) at 25°C. (g–k) Lifespan of genotypes described in (g) feeding on RNAi bacteria inhibiting *abu‐1* (i), *abu‐11* (j), both (k), or none (h) at 20°C. Rectangular insets describe statistics: Black asterisks or black ‘ns’ to the left of colored short lines indicate comparing to wildtype (WT); blue ‘ns’ indicates *p* ≥ 0.05 in log‐rank test if compared with *yyuEx70*; black asterisks or black ‘ns’ on top of two adjacent short lines or next to brace symbols, indicate comparisons between two referred groups represented by their corresponding short lines. Other statistical details are described in the legends of Figure 1.

Through qPCR analysis, we confirmed that in each temperature 6 of 8 *abu* genes reproduced their expression trends as in RNA‐seq (Figure 5b,c). When *nlp‐45* is absent, such 20°C‐specific upregulation of *abu* in the *miR‐80(Δ)* background is eliminated (Figure 5d; Figure S5d), while the 25°C‐specific downregulation is barely affected (Figure 5e; Figure S5 h,i). Then we respectively restored NLP‐45 expression in *nlp‐45(lf)* and *nlp‐45(lf)*; *miR‐80(Δ)*, and the *abu* levels generally recovered (Figure 5f; Figure S5 g–i). For completeness, we also examined the *abu* levels in animals lacking functional *egl‐3* or *unc‐31*, and results of which phenocopied *nlp‐45(lf)*'s effect (Figure S5a, b), while *nlp‐67(lf)* or *nlp‐25* RNAi or *nlp‐56* RNAi barely alters *abu* expression (Figure S5c, S5e, S5f). On the other hand, respective or combined RNAi of *abu‐1* or *abu‐11* strikingly weakened the life‐extending effects of *miR‐80(Δ)* and of *yyuEx70* (Figure 5h–k). Together, these results indicate that *miR‐80(Δ)* and NLP‐45 overexpression rely on the function of several ABU proteins to extend lifespan.

### 2.7 *miR‐80* *(Δ)* or NLP‐45 overexpression benefits longevity in the presence of moderate to strong ERS

**FIGURE 6:**
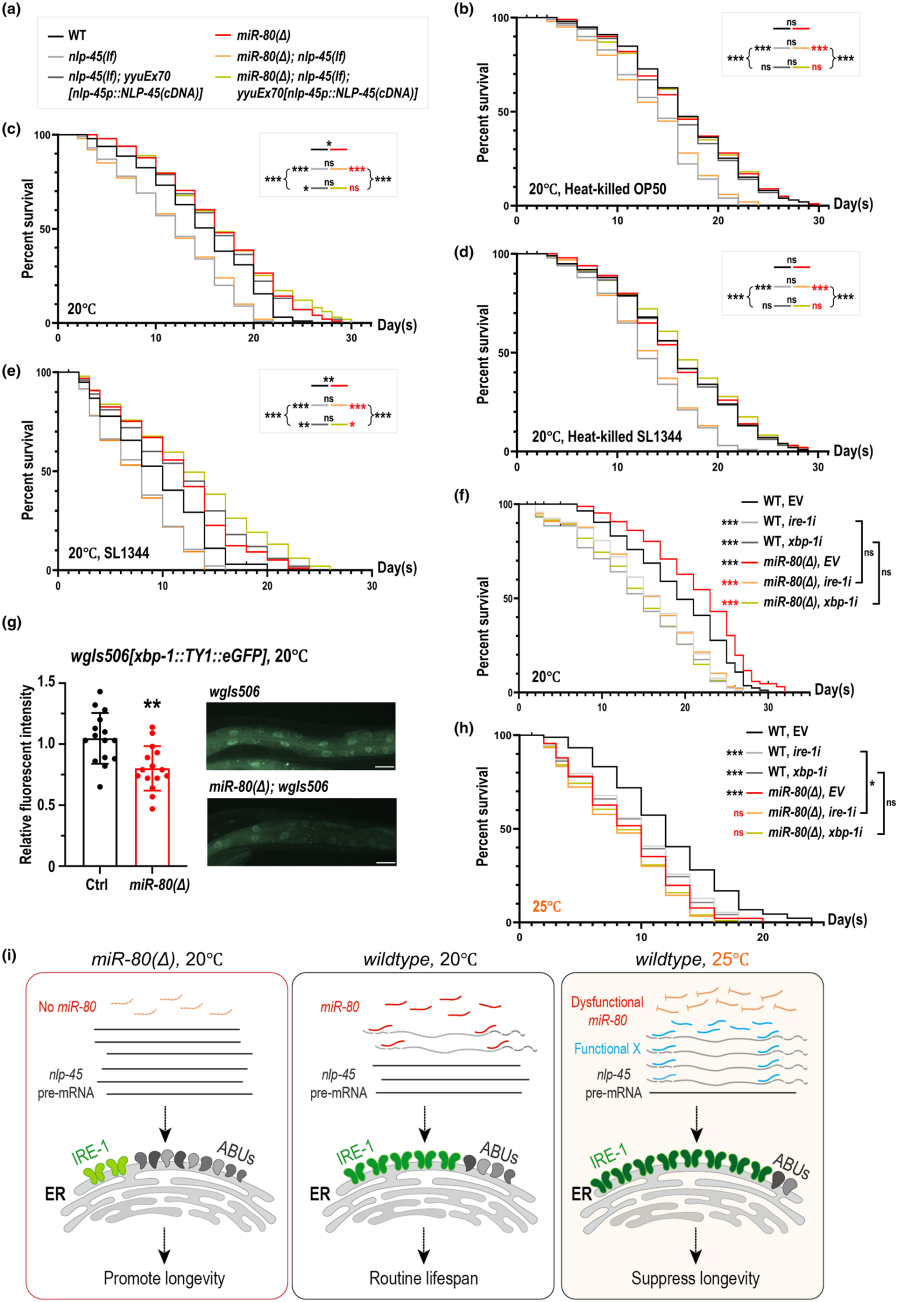
Lifespan extension of *miR‐80(Δ)* or NLP‐45 overexpression requires IRE‐1/XBP‐1. (a–f, h) Lifespan of genotypes described in (a) feeding on live OP50 (c, f), live SL1344 (e), heat‐killed OP50 (b), or heat‐killed SL1344 (d) at 20°C. Statistical symbols follow common rules: Black asterisks or black ‘ns’ next to short lines indicate comparing to wildtype; red asterisks or ‘ns’ next to short lines indicate comparing to *miR‐80(Δ)*; black asterisks or black ‘ns’ on top of two adjacent short lines or next to brace symbols, indicate comparisons between two referred groups represented by their corresponding short lines. (g) Quantified GFP intensity of a *xbp‐1* reporter, *wgIs506*, in wildtype and *miR‐80(Δ)* young adults at 20°C, with left panel for summarized mean and s.e. values and right panel for representative images (scale bar = 25 μm). Two asterisks represents Mann–Whitney test *p* < 0.01 under a null hypothesis assuming two groups are not different. Each dot represents an individual animal. (i) A model summarizing our findings in this study: At 20°C, *miR‐80* binds to the primary transcript of *nlp‐45* to restrain the level of NLP‐45, which leads to an IRE‐1‐dominant style for the handling of cellular ERS (middle), and removal of *miR‐80* benefits longevity via uninhibited NLP‐45 and increased involvedness of ABU proteins (left); at 25°C, *miR‐80* is sidelined or dysfunctional in terms of its weakened impact on NLP‐45, and *nlp‐45* could be downregulated by a mysterious factor X, which is associated with animals' reduced abu levels and relatively short lifespan. Other statistical details are described in the legends of Figure 1.

Inspired by the lifespan assays on killed bacteria for the studies of *abu* genes as in Haskins et al (Haskins et al., 2008), we wondered if aging attenuation of *miR‐80(Δ)* and NLP‐45 overexpression could be altered by the status of bacterial food, which generates ERS variations (Durai et al., 2014). When animals were cultured on ERS‐inducing conditions, for example, live SL1344 (*Salmonella enterica*) or tunicamycin treatment (Frakes et al., 2020; Haskins et al., 2008; Kim et al., 2010; Urano et al., 2002; Viswanathan et al., 2005), both *miR‐80(Δ)* and NLP‐45 overexpression managed to promote survival (Figure 6a,c,e). On the other hand, when animals were fed with heat‐killed bacteria, which provides ERS‐reduced environment (Durai et al., 2014), lifespan extension of *miR‐80(Δ)* or NLP‐45 overexpression no longer exists (Figure 6a,b,d), suggesting that *miR‐80(Δ)* or NLP‐45 overexpression only extends lifespan on the presence of moderate to strong ERS. Meanwhile, *nlp‐45(lf)* mutants remained short‐lived regardless of feeding states (Figure 6a–e; Figure S6d) or temperature (Figure 3e,f), indicating that a basal level of NLP‐45 is required for the maintenance of normal lifespan.

### 2.8 The IRE‐1/XBP‐1 pathway is indispensable for *miR*‐*80(Δ)* to extend lifespan at 20°C

Previous study has revealed that inactivated *abu‐1* led to enhanced intensity of hsp‐4::GFP, an IRE‐1/XBP‐1 pathway indicator (Urano et al., 2002). We wondered if *miR‐80(Δ)*, whose *abu* levels are upregulated 20°C‐specifically (Figure 5b), would conversely reduce hsp‐4::GFP. We compared the intensity of *zcIs4[hsp‐4::GFP]* (Bar‐Ziv et al., 2020; Calfon et al., 2002) for *miR‐80(Δ)* and wildtype. Indeed we observed a significant reduction of hsp‐4::GFP intensity in *miR‐80(Δ)* specifically at 20°C (Figure S6b,c), as confirmed by qPCR (Figure S6a). Moreover, at 20°C the fluorescent intensity of *wgIs506[xbp‐1::TY1::eGFP]* (Sarov et al., 2006) are reduced in *miR‐80(Δ)* (Figure 6g), while indicators of other UPR^ER^ pathways (Beaudoin‐Chabot et al., 2022) are not reduced (Figure S6a,d). Taking these results and the aforementioned publications together, we think the negative correlations between *abu* transcripts and IRE‐1/XBP‐1 activities might indicate tradable relationships for these noncanonical and canonical UPR^ER^ components to process stress.

Though levels of XBP‐1 and hsp‐4::GFP are relatively low in young adult *miR‐80(Δ)* at 20°C, we showed that the IRE‐1/XBP‐1 pathway is indeed indispensable for *miR‐80(Δ)* to ultimately attenuate aging, since *ire‐1* RNAi or *xbp‐1* RNAi would completely block the lifespan extension of *miR‐80(Δ)* at 20°C (Figure 6f). Although we do not exclude a possibility for *ire‐1i* or *xbp‐1i* to be generally detrimental, it is also reasonable for us to speculate that *miR‐80(Δ)* extends lifespan by increasing ABUs levels to protect IRE‐1/XBP‐1 from exhaustion, which explains *miR‐80(Δ)*'s efficiency at the presence of moderate or strong ERS as well as its inefficiency on low ERS conditions.

As NLP‐45 overexpression persistently alleviates aging at 25°C with or without *miR‐80* (Figure 3h), and reduced levels of *nlp‐45* and *abu* genes were found in *miR‐80(Δ)* at 25°C (Figures 3a and 5c), we tend to think that *miR‐80* per se functions in a temperature‐sensitive way, and the aging‐attenuating NLP‐45 could be regulated by alternative 25°C‐specific factors, sidelining *miR‐80*'s impact on *nlp‐45* (Figure 6i). At 25°C, age‐prone *miR‐80(Δ)* survived for comparably short periods with or without functional IRE‐1/XBP‐1 (Figure 6h), pointing to multiple possibilities for the role of *miR‐80* at this temperature, for example, *miR‐80* might inhibit unknown targets which are suppressors of IRE‐1/XBP‐1, or *miR‐80* and XBP‐1 might parallelly regulate a common downstream pathway, etc.

These findings collectively demonstrate a nuanced interplay between *miR‐80(Δ)*, NLP‐45, and UPR^ER^ components including the canonic IRE‐1/XBP‐1 pathway as well as the noncanonical ABU proteins, in *C. elegans* lifespan regulation. Our results highlight the unique roles of *miR‐80(Δ)* and NLP‐45 in conferring resistance to ERS, thereby contributing to the extension of lifespan under stressful conditions. This intricate mechanism underscores the potential of targeting *miR‐80*, NLP‐45, multiple ABU proteins, or other UPR^ER^ components in developing strategies aimed at lifespan modulation and stress resistance.

## 3 DISCUSSION

In this study, we present novel findings on the *miR‐80*/NLP‐45/UPR^ER^ axis in regulating *C. elegans* lifespan, with emphasis on the temperature‐dependency of a microRNA *miR‐80*. We revealed the 20°C‐specific lifespan‐extension of *miR‐80(Δ)* is achieved by upregulated NLP‐45, a neuropeptide‐like protein which may control the participation of distinct UPR^ER^ components toward ER stress. This study hopefully provides an example of genetic and environmental interplay in animals' lifespan determination.

Genes in the *miR‐58* family encode the most abundant microRNAs in *C. elegans*. Members of this family could have varied, and sometimes opposite effects on lifespan regulation, with complex and unexpected genetic interactions (Zhang et al., 2018). The *miR‐58*‐family seed region (7 nucleotides) is located at the 5′ end of *miR‐80*, while the other 16 nucleotides which do not belong to the seed region should be able to provide *miR‐80* unique roles, with the predicted binding of *miR‐80* and *nlp‐45* 5’‐UTR as a ready‐made case (Figure 4a). Mehul Vora et al. explained the mechanisms and effects of *miR‐80(Δ)* in extending lifespan under dietary restriction (Vora et al., 2013), and this study focuses on dissecting the lifespan extension of *miR‐80(Δ)* under normal dietary conditions. The most striking aspect of our study is the discovery of NLP‐45 as a key mediator in the *miR‐80* regulatory axis. Our investigation revealed that NLP‐45 is not merely a downstream target of *miR‐80*, but also plays a pivotal role in handling ERS, a critical aging‐accelerating factor.

Here, we constructed a simple model diagram to summarize our findings (Figure 6i): At 20°C, *miR‐80* binds to the primary transcripts of *nlp‐45* to restrict NLP‐45 at the basal level, associated by the dominancy of canonical IRE‐1/XBP‐1 pathway to process inevitably aging‐accompanied UPR^ER^ (middle panel); Deletion of *miR*‐80 permits abundant NLP‐45 and increased ABU proteins, which benefits survival by enhancing IRE‐1's durability (left panel); Shifting wildtype animals to mildly stressful 25°C disrupts *miR‐80*'s role by sidelining its impact on *nlp‐45* (right panel). Data of Figure 3a and Figure S3 strongly suggest that a mysterious 25°C‐specific X downregulates NLP‐45 in *miR*‐*80(Δ)*, yet we do not know how *miR*‐*80* and X may interact at variable temperatures. Importantly, we revealed that NLP‐45 overexpression firmly extends lifespan at both temperatures regardless of *miR‐80*'s presence, and we think in animals with NLP‐45 overexpression, increased ABU proteins might assist IRE‐1 to better‐process ER stress. Collectively, this novel study has connected lines for several dots which are either known or newly recognized regulators of longevity, including microRNAs, NLP, ABU proteins and the IRE‐1/XBP‐1 pathway.

NLP‐45 has been identified as an anti‐exploratory peptide, intricately regulated by a specific miRNA called *lin‐14* in the heterochronic pathway during the L1 to L2 developmental transition (Ewald et al., 2016). Our results indicate that *nlp‐45* might be a target of various factors (probably a target of various microRNAs), with *miR‐80* being just one of these key players. Interestingly, as day 1 or day 4 adults, *miR‐80(Δ)* displayed no additional improvement in pharyngeal pumping or body bend assays (Figure 1e,f), suggesting that the longevity‐benefiting effect of *miR‐80(Δ)* may become more pronounced as the organism ages. This phenomenon hints at the possibility that NLP‐45 or UPR^ER^ components could be subject to temporal regulatory variations, thereby exerting diverse biological functions. Disruptions of such regulatory networks have profound implications, as evidenced by studies linking similar mechanisms in mice, rats, or human beings to the development of cancer (Stasyk & Huber, 2016). This raises a critical question: do comparable miRNA‐mRNA mechanisms play roles in human aging? More research would be required for the full comprehension of parallels and distinctions in aging across different organisms.

While acute ER stress can activate protective mechanisms, chronic ER stress is often detrimental, leading to cell death and the progression of age‐related pathologies (De Lorenzo et al., 2023). The potential of *miR‐80* deletion or NLP‐45 overexpression to counteract lifespan shortening associated with UPR^ER^ components provides interesting working directions for the manipulation of chronic ER stress in future studies.

While our study establishes a link between *miR‐80*, NLP‐45 and UPR^ER^, it also opens avenues for further inquiry into the intricate molecular dynamics at play, such as analyzing mechanisms encoding *miR‐80*'s temperature dependency, identifying receptors and tissue‐specificity for NLP‐45, quantifying participation or durability of UPR^ER^ components, and so on. The fact that NLP‐45 levels do not strictly associate every *abu* gene's transcripts brings complexity to the understanding of relationships between *miR‐80*, NLP‐45 and ABU proteins. Meanwhile, we do not exclude the possibility for non‐NLP‐45 targets of *miR‐80* to regulate the transcriptional levels of *abu* genes. Revealing the direct or indirect regulation of NLP‐45 on the *abu* genes could unravel new aspects of stress‐responsive mechanisms. Determining the specific physiological or pathological conditions under which the *miR‐80*/NLP‐45/UPR^ER^ axis is activated will provide insights for the extension of health span.

In conclusion, this study offers a new perspective on the molecular mechanisms of the aging process. By linking lifespan‐regulating roles of miRNA, NLP and components processing ERS, our findings hopefully pave the way for novel therapeutic approaches to age‐related diseases and future methods to promote healthy aging in various organisms including human beings.

## AUTHOR CONTRIBUTIONS

C.Z. J.L. and Y.Y. conceived the study. C.Z. and Y.Z. performed the experiments. C.Z. and Y.Y. analyzed and interpreted the data and wrote the initial manuscript. C. Z., J. L. and Y.Y. wrote the revised manuscript. J.L. and Y.Y. obtained funding for this study.

## CONFLICT OF INTEREST STATEMENT

The authors declare no competing interests.

## Supporting information


Data S1.

Figure S1.
Figure S2.
Figure S3.
Figure S4.
Figure S5.
Figure S6.

Table S1.

Table S2.

Table S3.

## Data Availability

RNA‐seq data that support the findings of this study have been deposited in the NCBI Sequence Read Archive (SRA, http://www.ncbi.nlm.nih.gov/sra) under the BioProject ID PRJNA1058956. All other data supporting the findings of this study are available from the corresponding authors upon request.
