# Supplementary material for: Temperature‐dependent lifespan extension is achieved in miR‐80‐deleted Caenorhabditis elegans by NLP‐45 to modulate endoplasmic reticulum unfolded protein responses
Source: Aging Cell. 2024 Sep 25;24(1):e14345. doi: 10.1111/acel.14345 (PMC11709106; doi:10.1111/acel.14345)
Supplement: Supplementary file 1 — Data S1. [file ACEL-24-e14345-s005.docx]

**Supporting Information**

**Temperature-dependent lifespan extension is achieved in *miR-80*-deleted *Caenorhabditis elegans* by NLP-45 to modulate endoplasmic reticulum unfolded protein responses**

**Materials and Methods**

***Caenorhabditis elegans* strains and maintenance**

Unless otherwise mentioned, all *C. elegans* strains were maintained and propagated at 20°C on NGM agar seeded with *E. coli* OP50 using standard procedures (Stiernagle, 2006). All strains were outcrossed more than 4-times with N2 Bristol prior to use.

The following strains were obtained from *Caenorhabditis* Genetics Centre (CGC), University of Minnesota, USA: N2 Bristol (wild-type), MT15024 *miR-58(n4640)*, MT13949 *miR-80(nDf53)*, MT13954 *miR-81/miR-82(nDf54)*, VC461 *egl-3(gk238)*, DA509 *unc-31(e928)*, PS8027 *nlp-67(sy1176)*, OH16499 *nlp-45(ot1046)*, SJ4005 *zcIs4 [hsp-4::GFP]*, OH16380 *nlp-45(ot1032[nlp-45::T2A::GFP::H2B])*, OH16799 *otEx7681[mgl-1p::nlp-45 cDNA::SL2::TagRFP::p10 3’UTR + inx-6(prom18)::TagRFP]; nlp-45(ot1046)*, OP506 *unc-119(tm4063); wgIs506 [xbp-1::TY1::EGFP::3xFLAG + unc-119(+)]*, OP683 *unc-119(tm4063); wgIs683 [atf-6::TY1::EGFP::3xFLAG + unc-119(+)]*.

The following strains were gifted by Dr. Meng-Qiu Dong’s lab at National Institute of Biological Science, China: MQD1262 *miR-58(n4640); miR-80(nDf53)*, MT14128 *miR-80(nDf53); miR-81/miR-82(nDf54)*, MT15563 *miR-58(n4640); miR-80(nDf53); miR-81/miR-82(nDf54)*

The following mutant strains were generated in this study: YUY434 *miR-80(nDf53); egl-3(gk238)*, YUY437 *miR-80(nDf53); unc-31(e928)*, YUY484 *miR-80(nDf53); nlp-67(sy1176)*, YUY492 *miR-80(nDf53); nlp-45(ot1046)*, YUY540 nlp-45(ot1046); *yyuEx67[unc-119p::nlp-45 cDNA::SL2::GFP]*, YUY553 *miR-80(nDf53); nlp-45(ot1046); yyuEx67[unc-119p::nlp-45 cDNA::SL2::GFP]*, YUY689 *miR-80(nDf53); otEx7681; nlp-45(ot1046)*, YUY904 *miR-80(nDf53); nlp-45(ot1032[nlp-45::T2A::GFP::H2B])*, YUY905 *miR-80(nDf53); zcIs4 [hsp-4::GFP]*, YUY599 *miR-80(nDf53); nlp-45(ot1046); yyuEx70[nlp-45p::nlp-45 cDNA::SL2 GFP]*, YUY602 *nlp-45(ot1046); yyuEx70[nlp-45p::nlp-45 cDNA::SL2 GFP]*, YUY627 *miR-80(nDf53); nlp-45(ot1046); yyuEx73[nlp-45p::nlp-45 gDNA::3’UTR(-32bp)::SL2 GFP]*, YUY674 *nlp-45(ot1046); yyuEx73[nlp-45p::nlp-45 gDNA::3’UTR(-32bp)::SL2 GFP]*, YUY756 m*iR-80(nDf53); nlp-45(ot1046); yyuEx77[nlp-45p::5’UTR(-19bp)::nlp-45 gDNA::3’UTR(-32bp)::SL2 GFP]*, YUY759 *nlp-45(ot1046); yyuEx77[nlp-45p::5’UTR(-19bp)::nlp-45 gDNA::3’UTR(-32bp)::SL2 GFP]*, YUY762 *miR-80(nDf53); nlp-45(ot1046); yyuEx83[nlp-45p::5’UTR(-19bp)::nlp-45 gDNA::3’UTR::SL2 GFP*], YUY765 *nlp-45(ot1046); yyuEx83[nlp-45p::5’UTR(-19bp)::nlp-45 gDNA::3’UTR::SL2 GFP]*, YUY768 *miR-80(nDf53); nlp-45(ot1046); yyuEx89[nlp-45p::5’UTR::nlp-45 gDNA::3’UTR::SL2 GFP]*, YUY771 *nlp-45(ot1046);* *yyuEx89[nlp-45p::5’UTR::nlp-45 gDNA::3’UTR::SL2 GFP],* YUY993 *unc-119(tm4063); wgIs506 [xbp-1::TY1::EGFP::3xFLAG + unc-119(+)]; miR-80(nDf53)*, YUY994 *unc-119(tm4063); wgIs683 [atf-6::TY1::EGFP::3xFLAG + unc-119(+)]; miR-80(nDf53)*.

**Molecular cloning and transgenic strains**

The *nlp-45* overexpression strains were generated as below: primers were designed to amplify *C. elegans* cDNA or gDNA fragment of *nlp-45* with/without the predicted *miR-80* binding sites in the 5’/3’-UTR; promoters used in this study include the *unc-119p* as in pJM23-SL2-GFP (Vieira & Messing, 1987), and *nlp-45p,* a 2 kb region upstream of the transcription start site of *nlp-45*. Corresponding fragments were subsequently cloned into the pJM23-SL2-GFP backbone by seamless cloning to build distinct types of NLP-45 overexpression plasmids for the generation of *yyuEx67, yyuEx70,* and *yyuEx89*. Plasmids for *yyuEx73*, *yyuEx83* and *yyuEx77* were edited by commercial kits based on seamless cloning principles with the plasmid of *yyuEx89* as PCR templates. Standard *C. elegans* microinjection procedures (Rieckher & Tavernarakis, 2017) were applied, and all constructs were confirmed by Sanger sequencing before microinjection.

**Lifespan analysis**

Gravid adult hermaphrodites were synchronized in M9 buffer at 20°C overnight upon standard egg prep (Stiernagle, 2006), and then L1s were dropped onto OP50 seeded plates. After approximately 48 hours, 100 young adult animals were transferred to the lifespan assay plates in triplicates containing corresponding types of food lawns. For RNAi experiments, the RNAi bacteria were propagated from the Ahringer *C. elegans* RNAi feeding library (Kamath *et al*., 2003). Animals were then maintained at 20℃ or 25℃ and transferred every 2 days into fresh plates till the end of the reproductive period. Live worms were scored every 2 days, and dead worms were defined by not responding to a worm pick touch. Percents of live individuals were recorded every alternate day and plotted as % survival against the number of assay days. GraphPad Prism 8 (GraphPad Software) was used for log-rank tests and data plotting.

**Pharyngeal pumping and body bends**

Pharyngeal pumping and body bends were scored respectively as previously described (H. M. Kim *et al*., 2020). Roughly 15 nematodes were assayed in each group on different days of adulthood. Results were analyzed and plotted with GraphPad Prism 8 with the Mann-Whitney test.

**RT-qPCR analysis**

About 1000 individuals per genotype were harvested and used for total RNA isolation, and then a commercial kit (First-strand cDNA Synthesis Mix, LABLEAD) was used for the generation of cDNA templates. qPCR approach of miRNAs was modeled after Zhang *et al* (Zhang, Zhang, & Dong, 2018). miRNA expression levels were normalized to an endogenous control *U18*. Relative expression levels of *abu-1, abu-4, abu-6, abu-7, abu-8, abu-10, abu-11, abu-15, cbp-3, cht-1, hsp-4, aexr-1, flp-17, gnrr-3, nlp-4, nlp-16, nlp-25, nlp-30, nlp-34, nlp-45, nlp-56, nlp-67* and *tkr-3* mRNA were measured by qPCR using SYBR Green I according to manufacturer’s protocols (ROCHE), and normalized to the levels of *act-1* as an internal control. All qPCR primers were designed to span at least one exon-exon boundary. Statistics were analyzed by the *Welch’s t-*test in Microsoft Excel.

**RNA-seq and GO enrichment analysis**

For *miR-80(nDf53)* and wild-type respectively cultured at 20℃ and 25℃, four sets of same-day duplicates were prepared by harvesting ~2000 young adults per sample (duplicate), and then the experimental procedures of RNA-seq were entrusted to Novogene Company, Tianjin, China.

Upon routine quality control steps, sequencing data were proceeded as below: reads were aligned to *C. elegans* WBcel235 genome using Tophat2 (D. Kim *et al*., 2013); HTseq-count (Anders, Pyl, & Huber, 2015) was applied, and the counted values were imported to EdgeR (Robinson, McCarthy, & Smyth, 2010) for statistical analysis. For a certain gene, when the log_2_FoldChange is greater than 1 AND the adjusted *p* value as in EdgeR is smaller than 0.05, we marked it as an upregulated gene; when the log_2_FoldChange is smaller than -1 AND the adjusted *p* value as in EdgeR is smaller than 0.05, we marked it as a downregulated gene. Differential expression analysis and GO enrichment analysis were performed by Metascape (<https://metascape.org/>) (Zhou *et al*., 2019).

**Fluorescent microscopy**

Transgenic animals with *zcIs4[hsp-4::GFP]* or *wgIs506[xbp-1::TY1::eGFP]* or *wgIs683[atf-6::TY1::eGFP]* at young adult stage were anesthetized in 1% sodium azide in M9 buffer and placed on 2% agarose pads sandwiched between coverslips and glass slides under an inverted microscope (Zeiss Axioplan 2) with 20x objective lens and monochromatic camera. No less than 15 individuals were imaged, and the ImageJ software was used for area-based quantification.

Fluorescence of *ot1032[nlp-45::T2A::GFP::H2B]* was quantified with the same methods mentioned above, except that additional animals were harvested on the 1st, 4th, and 7th days of adulthood, which are achieved by similar procedures as in lifespan assays.

**References**

Anders, S., Pyl, P. T., & Huber, W. (2015). HTSeq--a Python framework to work with high-throughput sequencing data. *Bioinformatics, 31*(2), 166-169. doi:10.1093/bioinformatics/btu638

Kamath, R. S., Fraser, A. G., Dong, Y., Poulin, G., Durbin, R., Gotta, M., . . . Ahringer, J. (2003). Systematic functional analysis of the *Caenorhabditis elegans* genome using RNAi. *Nature, 421*(6920), 231-237. doi:10.1038/nature01278

Kim, D., Pertea, G., Trapnell, C., Pimentel, H., Kelley, R., & Salzberg, S. L. (2013). TopHat2: accurate alignment of transcriptomes in the presence of insertions, deletions and gene fusions. *Genome Biol, 14*(4), R36. doi:10.1186/gb-2013-14-4-r36

Kim, H. M., Long, N. P., Min, J. E., Anh, N. H., Kim, S. J., Yoon, S. J., & Kwon, S. W. (2020). Comprehensive phenotyping and multi-omic profiling in the toxicity assessment of nanopolystyrene with different surface properties. *J Hazard Mater, 399*, 123005. doi:10.1016/j.jhazmat.2020.123005

Rieckher, M., & Tavernarakis, N. (2017). *Caenorhabditis elegans* Microinjection. *Bio Protoc, 7*(19). doi:10.21769/BioProtoc.2565

Robinson, M. D., McCarthy, D. J., & Smyth, G. K. (2010). edgeR: a Bioconductor package for differential expression analysis of digital gene expression data. *Bioinformatics, 26*(1), 139-140. doi:10.1093/bioinformatics/btp616

Stiernagle, T. (2006). Maintenance of *C. elegans*. *WormBook*, 1-11. doi:10.1895/wormbook.1.101.1

Vieira, J., & Messing, J. (1987). Production of single-stranded plasmid DNA. *Methods Enzymol, 153*, 3-11. doi:10.1016/0076-6879(87)53044-0

Zhang, Y., Zhang, W., & Dong, M. (2018). The *miR-58* microRNA family is regulated by insulin signaling and contributes to lifespan regulation in *Caenorhabditis elegans.* *Sci China Life Sci, 61*(9), 1060-1070. doi:10.1007/s11427-018-9308-8

Zhou, Y., Zhou, B., Pache, L., Chang, M., Khodabakhshi, A. H., Tanaseichuk, O., . . . Chanda, S. K. (2019). Metascape provides a biologist-oriented resource for the analysis of systems-level datasets. *Nat Commun, 10*(1), 1523. doi:10.1038/s41467-019-09234-6
