# Supplementary material for: Temperature‐dependent lifespan extension is achieved in miR‐80‐deleted Caenorhabditis elegans by NLP‐45 to modulate endoplasmic reticulum unfolded protein responses
Source: Aging Cell. 2024 Sep 25;24(1):e14345. doi: 10.1111/acel.14345 (PMC11709106; doi:10.1111/acel.14345)
Supplement: Supplementary file 2 — Figure S1. Figure S2. Figure S3. Figure S4. Figure S5. Figure S6. [file ACEL-24-e14345-s004.pdf]

Supplementary Figure 1

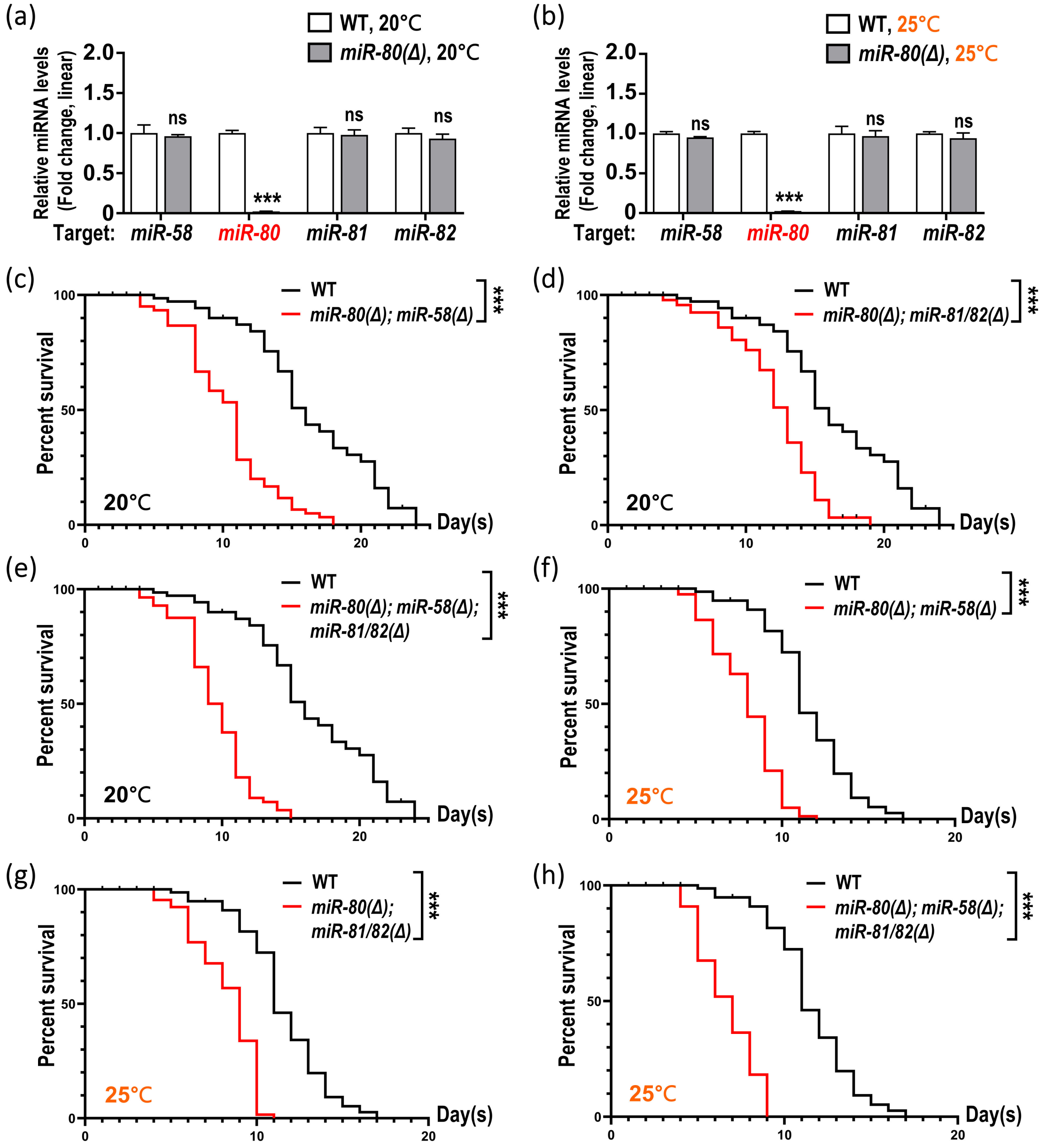


**Supplementary Fig. 1 Deletion of *miR-80* does not alter *miR-58*, *miR-81* and *miR-82* levels, and fails to extend lifespan in mutants lacking functional *miR-58*, *miR-81* and/or *miR-82*** **(a-b)** Levels of *miR-58*, *miR-81*, and *miR-82* remain unchanged in *miR-80(Δ)* at 20°C **(a)** or 25°C **(b)**. **(c-h)** Lifespan assays of double or triple mutants involving corresponding combinations of *miR-58(Δ)*, *miR-80(Δ)*, *miR-81/82(Δ)* at 20°C **(c-e)** or 25°C **(f-h).** Statistics are described in the legends of Fig. 1.

Supplementary Figure 2

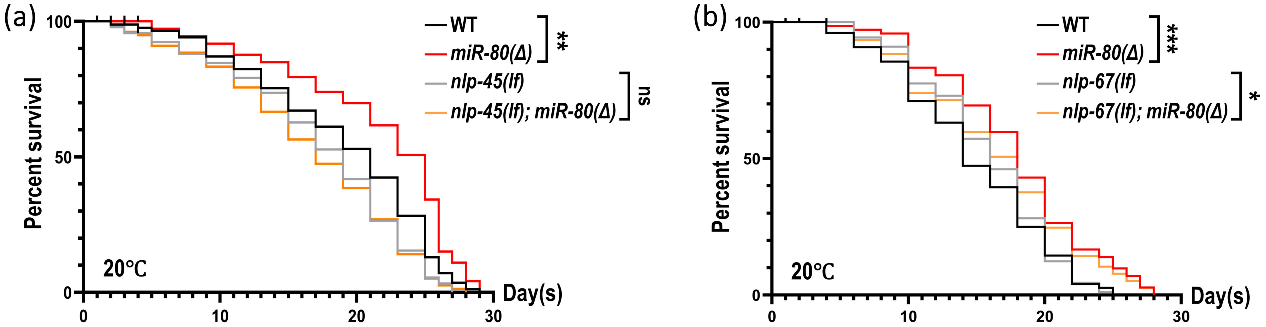


**Supplementary Fig. 2 Lifespan-extension of *miR-80(Δ)* is blocked in *nlp-45(lf)* but not *nlp-67(lf)***

**(a-b)** Lifespan assays with animals lacking functional *miR-80, nlp-45* (**a**) or *nlp-67* (**b**), or both, at 20°C. Statistics are described in the legends of Fig. 1.

Supplementary Figure 3

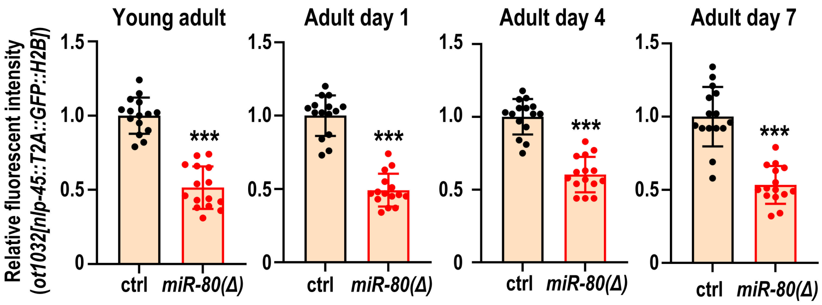


**Supplementary Fig. 3 Expression of NLP-45 is decreased in *miR-80(Δ)* at 25°C**

In the young adult stage and on the 1st, 4th, and 7th day of adulthood, GFP intensities of *ot1032[nlp-45::T2A::GFP::H2B])* were imaged and compared in between *miR-80(Δ)* and *wildtype* at 25°C. Columns indicate the mean values, and error bars indicate s.e.. Three asterisks indicate $p < 0.001$ for the Mann-Whitney test under a null hypothesis assuming two groups are not different. Each dot represents an individual animal.

Supplementary Figure 4

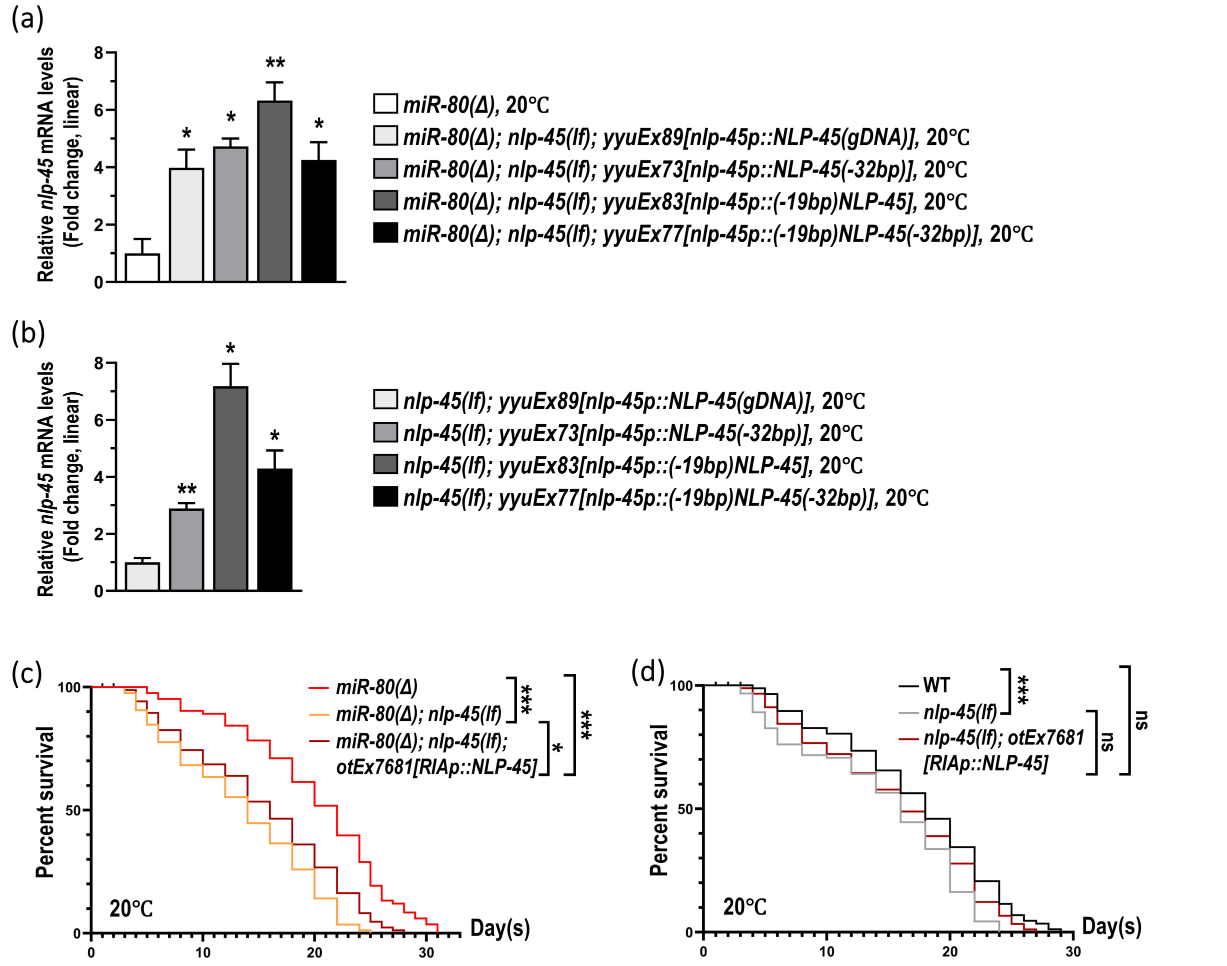


**Supplementary Fig. 4 *miR-80* directly regulates NLP-45 by binding to its UTRs**

(**a-b**) Relative transcript levels of qRT-PCR on *nlp-45* genes in *nlp-45(lf)* backgrounds with or without *miR-80.* When *miR-80* is absent, the *nlp-45* transcripts of *yyuEx89*, *yyuEx73*, *yyuEx83*, or *yyuEx77* are significantly greater than that of the control (**a**); When *miR-80* is present, the *nlp-45* transcripts of *yyuEx73*, *yyuEx83*, or *yyuEx77* are significantly greater than that of *yyuEx89*, meaning that *miR-80*'s suppression on *nlp-45* has been weakened with impaired *nlp-45* UTR (**b**). (**c-d**) Lifespan assays with RIA-specific overexpression of *nlp-45* in *miR-80(Δ)* (**c**) or *wildtype* (**d**). Other statistical information is described in the legends of Fig. 1.

Supplementary Figure 5

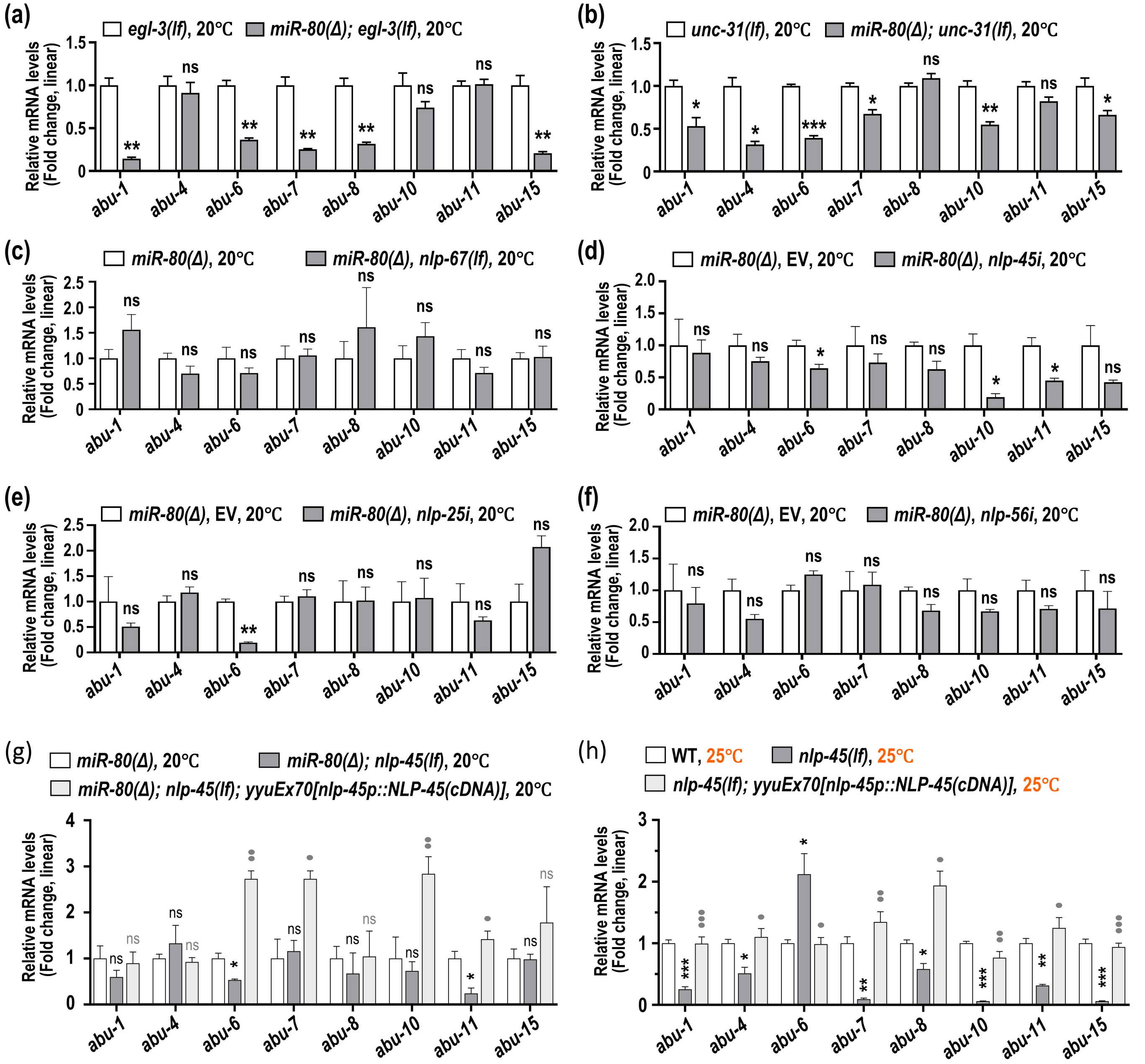


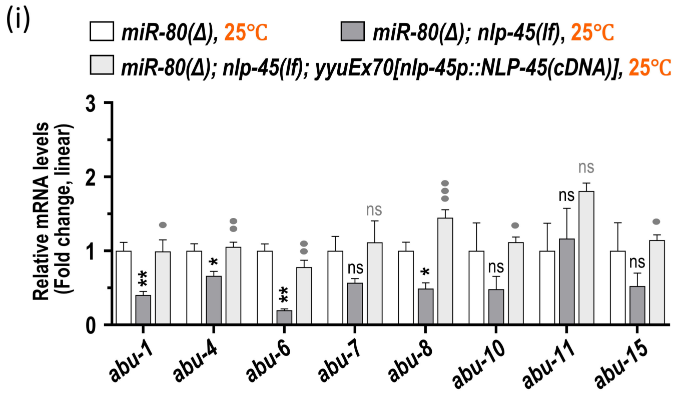


**Supplementary Fig. 5 UPR target genes are regulated by *miR-80*.**
**(a,b)** Relative transcript levels of qRT-PCR on *abu* genes for templates with or without *miR-80* in the background of *egl-3(lf)* (**a**) or *unc-31(lf)* (**b**) at 20°C. (**c**) Relative transcript levels of qRT-PCR on *abu* genes for templates with or without *nlp-67* in *miR-80(Δ)* at 20°C. (**d-f**) Relative transcript levels of qRT-PCR on *abu* genes for *miR-80(Δ)* templates feeding on control bacteria or the RNAi bacteria suppressing *nlp-45* (**d**), *nlp-25* (**e**), *or nlp-56* (**f**) at 20°C. (**g**) Relative transcript levels of qRT-PCR on *abu* genes for *miR-80(Δ)*, *miR-80(Δ); nlp-45(lf)*, and *miR-80(Δ); nlp-45(lf); yyuEx70* at 20°C. (h-i) Relative transcript levels of qRT-PCR on *abu* genes for animals bearing control, *nlp-45(lf)*, and *nlp-45(lf); yyuEx70* in the background of *wildtype* (**h**) or *miR-80(Δ)* (**i**) at 25°C. Statistics are described in the legends of Fig. 1 and Fig. 5.

Supplementary Figure 6

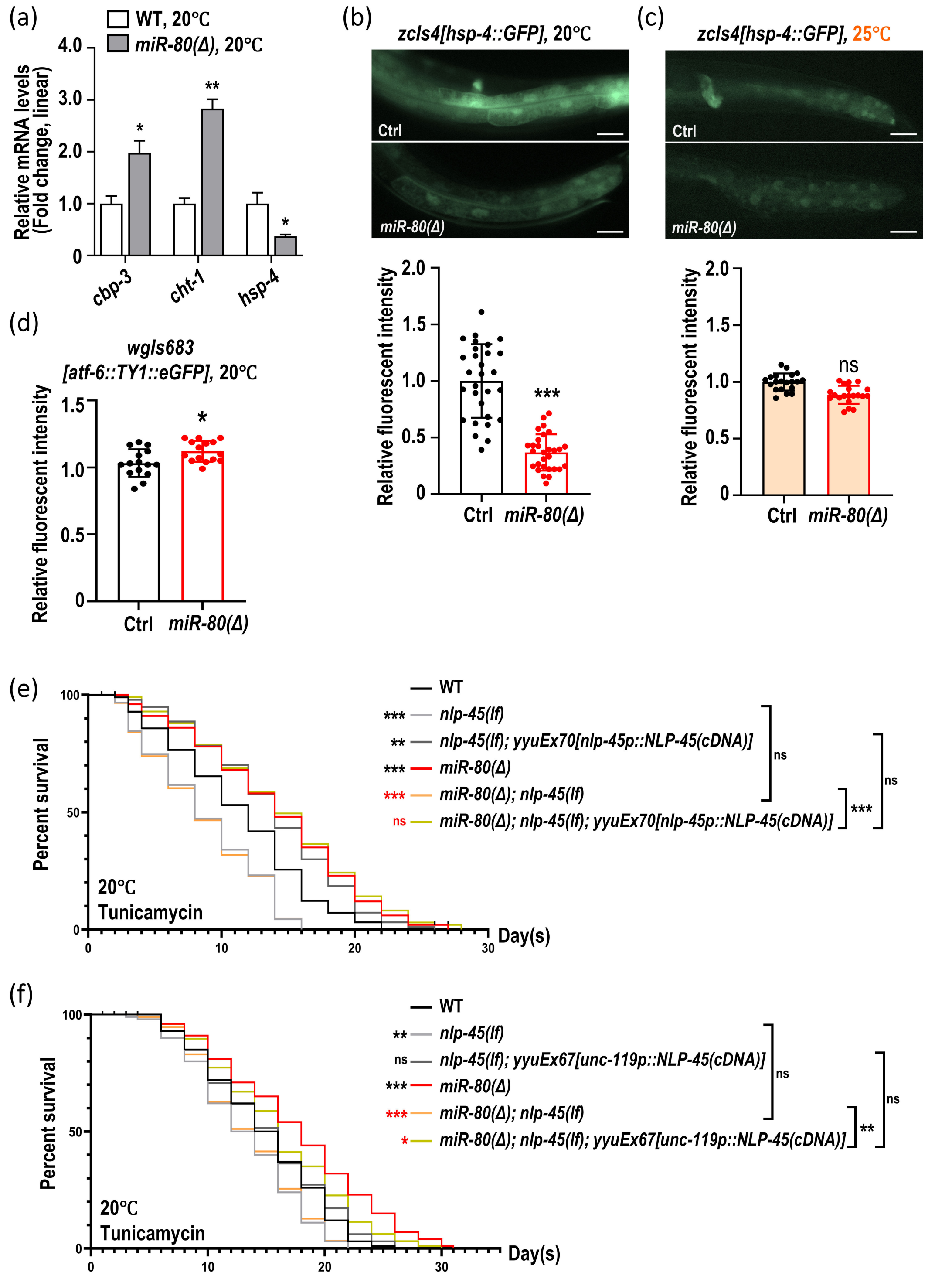


**Supplementary Fig. 6 Different UPR$^{ER}$ components exhibit distinct roles in *miR-80*(Δ)-mediated lifespan extension**

**(a)** Relative transcript levels of qRT-PCR on *cbp-3*, *cht-1* and *hsp-4* in *wildtype* and *miR-80(Δ)* at 20°C. **(b-c)** GFP intensities of *zcIs4* in *wildtype* and *miR-80(Δ)* young adults at 20°C (**b**) or 25°C (**c**). The Mann-Whitney tests were applied under a null hypothesis assuming two groups are not different and mean with s.e. values were plotted. Each dot represents an individual animal. Scale bar = 25 µm. **(d)** GFP intensities of *wgIs683* were imaged and compared between *miR-80(Δ)* and *wildtype* at 20°C. Mean with s.e. values were plotted, each dot represents one individual. One asterisk indicates that $p < 0.05$ for the Mann-Whitney test under a null hypothesis assuming two groups are not different. **(e-f)** Lifespan assays with animals treated by 5 mg/mL tunicamycin. With endogenous promoter, NLP-45 overexpression extends lifespan to a *miR-80*(Δ)-like level (**e**); Pan-neural overexpression of NLP-45 in the *miR-80(Δ); nlp-45(lf)* partially restores the lifespan (**f**). Other statistical information is described in the legends of Fig. 1.
